# A systematic review of people’s lived experiences of inpatient treatment for anorexia nervosa: living in a “bubble”

**DOI:** 10.1186/s40337-023-00820-0

**Published:** 2023-06-09

**Authors:** Rebekah Rankin, Janet Conti, Lucie Ramjan, Phillipa Hay

**Affiliations:** 1 Translational Health Research Institute, School of Medicine, Western Sydney University, Sydney, Australia; 2 School of Psychology, Western Sydney University, Sydney, Australia; 3 School of Nursing and Midwifery, Western Sydney University, Sydney, Australia; 4 Campbelltown Hospital, South West Sydney Local Health District (SWSLHD), Sydney, Australia

**Keywords:** Eating disorder, Anorexia nervosa, Inpatient treatment, Systematic review, Meta-synthesis, Qualitative, Therapy, Intervention, Refeeding, Lived experience

## Abstract

**Background:**

Treatment for anorexia nervosa (AN) is typically delivered on a continuum of care, starting with outpatient treatment, and moving onto intensive outpatient, day or residential treatment and/or inpatient hospitalisation. However, minimal attention has been afforded to the lived experiences of persons undergoing inpatient treatment for AN. In particular, qualitative literature pertaining to the lived experiences of specialist inpatient or residential treatment of AN remains fragmented and incomplete. The aim of this review was to synthesise current literature exploring patients’ lived experiences of residential and inpatient treatment for AN within eating disorder-specific treatment services.

**Methods:**

Five databases were searched and a qualitative thematic systematic review and meta-synthesis of 11 studies were conducted.

**Results:**

Eleven studies of 159 participants were included. Four meta-themes were constructed from the data: (1) a medical discourse—“I don’t think it’s individualised here”; (2) restrictive practice—living in a “bubble”; (3) myself, others and “a similar demon”; and (4) I am “not just another anorexic”. The data also revealed two cross-cutting themes: (1) more than a single experience; and (2) meaning making and identity.

**Conclusions:**

These findings highlight the complex and multifaceted nature of the inpatient treatment experience as well as the inherent conflicts in balancing the necessity of medical and psychological intervention with person-centred treatment approaches in the treatment of AN.

**Supplementary Information:**

The online version contains supplementary material available at 10.1186/s40337-023-00820-0.

## Abstract (plain language summary)

Treatment for anorexia nervosa (AN) is typically delivered on a continuum of care, starting with outpatient treatment, and moving onto intensive outpatient, day or residential treatment and/or inpatient hospitalisation. However, not much is known about the lived experiences of people going through inpatient treatment for AN. An improved understanding of individuals’ treatment experiences is essential in informing the development of new models of care with the potential to improve outcomes. In this systematic review, we examined the lived experiences of individuals going through inpatient treatment for AN available in the current literature. Searching in scientific databases resulted in 10,666 articles, 11 of which met the rigorous inclusion criteria. Four meta-themes and two cross-cutting themes were observed in patient experiences across the studies reviewed. These themes provide insight into the complex and multifaceted nature of the inpatient treatment experience as well as the inherent conflicts in balancing the necessity of medical and psychological intervention with person-centred treatment approaches in the treatment of AN.

**Supplementary Information:**

The online version contains supplementary material available at 10.1186/s40337-023-00820-0.

## Background

Anorexia nervosa (AN) is a serious, complex and potentially life-threatening psychiatric illness, characterised by low body weight, body-image distortion and an intense fear of gaining weight [1, 2]. This illness is indiscriminate—affecting individuals of all ages, genders, ethnicities, socioeconomic backgrounds and body weights—and ranges in both complexity and severity [3]. Based on epidemiological research, the mean-weighted lifetime prevalence rate of AN is 1.4% (range 0.1–3.6%) in women and 0.2% (range 0–0.3%) in men [4]. People with AN also experience lower levels of employment participation, high healthcare costs and early mortality [5–7].

Treatment for AN is typically delivered on a continuum of care, starting with outpatient treatment, and moving onto intensive outpatient, day or residential treatment, and/or inpatient hospitalisation [8]. An individual’s treatment journey through the various levels of care is unique, constantly changing and dependent on numerous factors such as treatment availability, patient motivation, and treatment history, symptom severity, medical stability, residential location and financial constraints [11]. Higher levels of care (e.g., day, residential treatment, and inpatient treatments) are reserved for those who are medically compromised and/or unable to be effectively treated in outpatient or community treatment settings [8, 9]. Historically, hospital treatment programs for AN have focused on refeeding and medical stabilisation, applying a didactic ‘one-size-fits-all’ model of care, with community treatment teams only being developed within psychiatric or psychosomatic settings in the latter part of the twentieth century [10]. Despite ongoing advancements in the understanding of AN, research indicates that optimal care management has yet to be realised [8, 11–13]. Long-term (10–20 years) follow-up studies [13–15] of those who have received inpatient treatment for eating disorders found that between 60 and 64% of individuals previously diagnosed with AN still met diagnostic criteria for an eating disorder at follow-up. While many people with severe and enduring AN are labelled as ‘treatment resistant’ [16, 17], it is also possible that this group of individuals have simply been unable to access treatment that is suited to their unique needs and preferences that may support recovery [18]. These perceived deficiencies in care have driven alternate and more collaborative models of care with a much greater representation of people with lived experience on the treating team, such as the Carolyn Costin Monte Nido residential programs, which include the use of lived-experience peer mentors and clinical staff who have a lived experience of an eating disorder as a core component of their programs [19, 20].

Most research regarding inpatient and residential treatment for AN has been quantitative and focused on treatment outcomes, measured mainly by ED symptoms or specific treatment factors such as the delivery of a particular intervention in an inpatient setting [20–22]. As such, minimal attention has been afforded to the lived experiences of persons undergoing inpatient treatment for AN. This is an important gap as improved understanding is essential to inform the development of new models of care with the potential to improve outcomes. While a small number of systematic reviews and meta-syntheses of the qualitative literature pertaining to the lived experience of eating disorder treatment and recovery exist [23–25], these reviews examine individuals’ treatment experiences across multiple treatment settings or focus on patients’ experiences of involuntary treatment [26]. Qualitative literature pertaining to the lived experiences of specialist inpatient or residential treatment of AN remains fragmented and incomplete.

To our knowledge there are no other published systematic reviews of qualitative literature regarding the lived experience of participants in eating disorder-specific treatment facilities that were inpatient specialist and/or residential. Thus, this paper aims to conduct a meta-synthesis of current literature pertaining to patients’ lived experiences of residential and inpatient treatment for AN within eating disorder-specific treatment services. This will provide a greater understanding of the contemporary literature and inform future research and interventions.

## Methods

### Study design

This meta-synthesis relies on the model of meta-ethnography and follows the procedures of the thematic synthesis described by Thomas and Harden [27]. In compliance with the Enhancing Transparency in Reporting the Synthesis of Qualitative Research (ENTREQ) guidelines [28], this synthesis consisted of six stages: (1) defining the research question, the subjects and the types of studies to be included; (2) identifying and selecting the studies; (3) assessing the quality of the selected studies; (4) analysing the studies, identifying their themes and translating these themes across the studies; (5) generating meta-themes and structuring the synthesis; and (6) writing the synthesis findings. The thematic analysis contained two phases: one descriptive, which defined and compared the themes, and the other interpretive, which developed original ideas drawn from the review.

### Search strategy

This systematic review search was conducted in accordance with the updated Preferred Reporting Items for Systematic Reviews and Meta-Analyses (PRISMA) guidelines [29]. The protocol was approved by PROSPERO (ID number: CRD42023349066). A systematic electronic search of PubMed, PsychINFO, SCOPUS, Web of Science and ProQuest Psychology databases was conducted by the first author (RR) on April 27, 2023, following consultation with a health librarian. The following search terms and Boolean operators were employed where the terms appeared in either the title or abstract of the article: “eating disorder*” OR “ano-rex*” OR “anore*” AND “intervention*” OR “treat*” OR “residential*” OR “partial hospitalization” OR “inpatient*” AND “qualitative*” OR “perspective*” OR “experience*”. Given the shifts in inpatient treatment approaches in the twentieth century [12], the authors elected to focus on contemporary lived experiences of inpatient or other residential care. To capture patients’ lived experiences of eating disorder-specific treatment services, the search was limited to human subjects and articles published in peer-reviewed journals in the English language between January 2010 and April 2023.

### Study selection

All search outputs were cross-referenced, and duplicate records removed using Covidence [30]. Screening of titles and abstracts was shared between authors to identify articles likely to be eligible. Full texts of identified studies were reviewed by two authors (RR, JC) to determine if studies met the following inclusion criteria: (a) qualitative research design or presented qualitative findings as a part of a mixed-methods design; (b) focused exclusively on the experience of inpatient and/or residential treatment for AN in an eating disorder-specific treatment setting; (c) all participants formally met *Diagnostic and Statistical Manual of Mental Disorders* (5th Edition) (DSM-5) [1] or *World Health Organisation International Statistical Classification of Diseases and Related Health Problems* (ICD-10) [2] diagnostic criterion for AN at the time of seeking treatment; and (d) published in a peer-reviewed journal.

Studies were excluded for the following reasons: (a) participants received inpatient treatment for a diagnosis other than an AN diagnosis; (b) treatment was provided in an outpatient or non-specified treatment setting; (c) the study focused on a specific treatment or intervention (e.g., family-based treatment) rather than the inpatient or residential experience; (d) the qualitative data presented were minimal (e.g., there were no extracts); (e) mixed or non-eating disorder-specific treatment setting; (f) published in a language other than English; and (g) was not published in a peer-reviewed journal. Any discrepancies in study selection were noted and resolved through discussion with a third author (LR). The final inclusion of the articles was based on consensus amongst three authors (RR, JC, LR).

### Quality assessment

Articles were assessed by three authors (RR 100%; JC 50%; LR 50%) following guidance provided by the Critical Appraisal Skill Programme (CASP) [31] tool for qualitative research studies. The purpose of the tool is not to provide an absolute score of quality, but rather to facilitate consideration of clarity of aims, appropriateness of methods, design and recruitment methods, suitability of data collection, researcher reflexivity, ethics, analytic rigour and clarity of findings. Given the lack of consensus regarding the role and function of study quality in systematic reviews [32, 33], for the purpose of the current review no papers were excluded based on their quality assessment scores. However, in keeping with the meta-ethnographic approach, studies of poorer quality contributed less to the synthesis. An outline of the quality assessment for each study can be found in Additional file 1: Table A.

### Data extraction and synthesis

Our analysis followed the procedure described by Sattar et al. [32] and Thomas and Harden [27], adapting them to the principles of the meta-ethnographic approach [32, 34]. It began with an attentive reading and then repeated readings of the titles, abstracts and texts of each article. One author (RR) extracted the formal characteristics of the studies, and extracted and analysed the first-order results (that is, the qualitative extracts using pseudonyms chosen by original papers) and the second-order results (authors’ interpretations and discussions of the results) of each study using a custom template in Covidence [30]. No additional data were requested from the original investigators.

NVivo 12 [35] qualitative analysis software was used to manage all data and facilitate the generation of themes. Extracted data and analyses were coded and a set of themes and subthemes inductively developed (RR) according to Tomas and Harden’s [27] three-phase approach to thematic synthesis. Coding notes were collaboratively (JC; LR; PH; RR) reviewed for overlapping and repetitive codes by the research team prior to being collapsed into subthemes. Similar subthemes were clustered together to delineate major patterns in the data, creating overarching themes. Differences of opinion were resolved through discussion. Final themes were selected by all authors (JC; LR; PH; RR) and extracts embedded within the analytic narrative to create a coherent and meaningful representation of participants’ lived experience of inpatient or residential treatments for AN [36]. Researcher position statements are provided (see Additional file 2).

## Results

### Presentation of studies

**Fig. 1:**
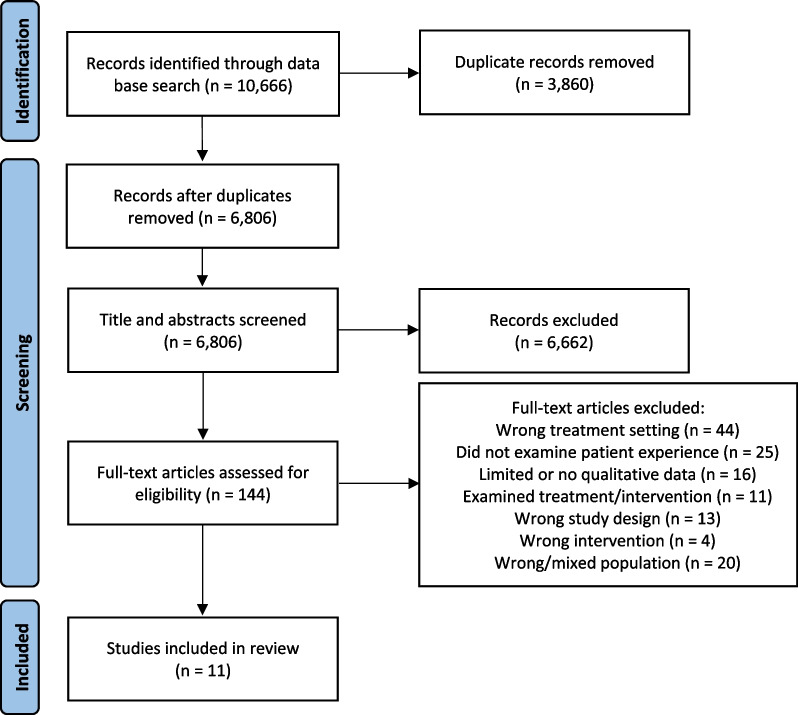
Flow of selecting and excluding studies according to the PRISMA guidelines for systematic reviews

As outlined in Fig. 1, the initial search identified a total of 10,666 articles—6806 following the removal of duplicates. Following title and abstract screening, 144 articles were selected for full text review. Of these, 11 met full inclusion criteria. The authors note that all studies were conducted in inpatient public and private settings. No publications qualitatively exploring the lived experience of individuals undergoing treatment for AN in residential settings were identified.

From the 11 papers that met the search criteria, there were 159 participants (156 female; 3 male) ranging in age from 12 to 45 years. All studies included adolescent (age ≤ 18) participants, with four studies focusing solely on adolescent inpatient treatment experiences. Illness duration varied and was only reported in four studies. The included studies were conducted in five different countries: United Kingdom (n = 5); Australia (n = 2); New Zealand (n = 1); Israel (n = 1); Norway (n = 1); and Denmark (n = 1). Data were predominantly collected through semi-structured or in-depth interviews. Qualitative methods used among the studies included thematic analysis, grounded theory, interpretative phenomenological analysis, narrative analysis, discourse analysis and autoethnography. Table 1 provides a descriptive summary of the studies included in this review.

**Table 1:** Characteristics of studies included

Author	Year	Aim	Country	Setting	Population	Diagnoses and illness duration (ID)	Recruitment	Data collection	Method
Malson et al. [37]	2004	To analyse participants’ accounts of their in-patient treatment experiences, through explicating the ways in which ‘the eating disordered patient’ is constituted as a subject position or imposition in these accounts	United Kingdom; Australia	IP specialist ward UK; General adolescent ward AUS	Participants n = 39 Age 14–45 years Female (n = 39)	Diagnoses: AN; BN ID = not specified	Medical service admission during time point	SSI during admission & post discharge	DA
Colton & Pistrang [38]	2004	To provide a detailed description of how adolescents experience inpatient treatment for anorexia nervosa	United Kingdom	IP specialist unit; 10 bed milieu setting	Participants n = 19 Age 12–17 to years (M = 15.4 years) Female (n = 19)	Diagnosis: AN ID: M = 23 months (SD 12.4 months) from diagnosis	Medical service admission during time point	Narrative SSI with member checking; post discharge	IPA
Boughtwood and Halse [39]	2010	To identify how teenage girls diagnosed with anorexia construct their illness, treatment programme and relationships with their doctors and nurses	Australia	IP hospital setting (not specified)	Participants n = 25 Age 12–18 years Female (n = 25)	Diagnosis: AN ID = not specified	Voluntary; Invitation to all participants admitted to medical service during specified period	SSI interviews and researcher field notes; During admission (n = 20) & post discharge (n = 5)	Discourse analysis (post-structural theory framework)
Long et al. [40]	2011	To investigate in-patient perceptions of mealtimes on eating disorders units	United Kingdom	IP eating disorder unit × 3; independent eating disorder service × 1	Participants n = 12 Age M = 22 years (SD 3.74 years) Female (n = 12)	Diagnosis: AN ID = not specified	Invitation sent post discharge to all participants admitted to medical service during specified period	Interview, open ended questions following: "tell me about your experiences of mealtimes as an in-patient"; Post discharge	TA
Eli [41]	2014	T0 identify the ways in which inpatient ambivalence might be embedded in the special social institutional setting that an eating disorders ward presents, beyond patient-specific motivation for recovery	Israel	IP specialist ward	Participants n = 13 Age 18–38 years Female (n = 12) Male (n = 1)	Diagnosis: AN = 12; BN = 1 ID = not specified	Multiple sources: outpatient eating disorders clinic; online pro-recovery eating disorders forum; eating disorders advocacy organization, and; chain-referral/snowball sampling	SSI (narrative focus) following discharge	IPA (contextual framework)
Kezelman et al. [42]	2016	To provide a detailed qualitative analysis of an individual’s psychological experience across the course of an inpatient treatment implementing rapid-refeeding protocol	Australia	IP specialist adolescent medical	Participants n = 10 Age = 17–19 years Female (n = 10)	Diagnosis: AN ID: M = 10.1 months (SD 11.7 months) from diagnosis	Invitation to all participants admitted to medical service during specified period	3 × weekly SSI during admission	Thematic analysis
Smith et al. [43]	2016	To explore experiences of women undergoing specialist inpatient treatment for anorexia nervosa	Scotland	IP specialist unit	Participants n = 21 Age 18–41 years Female (n = 21)	Diagnosis: AN ID: M = 76.1 months (SD = 86.3 months) subjective report	Voluntary; Invitation to all participants admitted to medical service during specified period	SSI at or following discharge	TA (Inductive; realist approach)
Thabrew et al. [44]	2020	To: (i) Understand the experiences of young people with anorexia admitted to hospital for briefer stays; and (ii) inform the design of contemporary inpatient treatment to better suit their needs	New Zealand	IP tertiary specialist eating disorder service	Participants n = 9 Age 15–17 years Female (n = 8) Male (n = 1)	Diagnosis: AN ID = 1–24 months from diagnosis	Invitation to all participants admitted to medical service during specified period	SSI post discharge	TA
Solhaug and Alsaker [45]	2021	To explore patient lived experience of inpatient/hospital treatment	Norway	IP specialist unit	Participants n = 3 Age 18–30 years Female (n = 2) Male (n = 1)	Diagnosis: AN ID = not specified	Voluntary; Invitation to all participants admitted to medical service during specified period	Patient diaries (responses to open ended questions) during treatment	Thematic interpretative analysis
MacDonald et al. [46]	2023	Living and leaving a life of coercion: a qualitative interview study of patients with anorexia nervosa and multiple involuntary treatment events	Denmark	Multiple IP specialist settings	Participants n = 7 Age 20–40 years Female (n = 7)	Diagnosis: AN ID = not specified. All participants had > 5 inpatient admissions	Purposeful sampling through flyers and social media, shared by/displayed at specialised treatment facilities and national eating disorder organization	SSI post discharge	Reflexive TA
O’Connell [47]	2023	Being and doing anorexia nervosa: An autoethnography of diagnostic identity and performance of illness	United Kingdom	IP adult specialist unit	Participants n = 1 Age not specified Female (n = 1)	Diagnosis: AN ID = not specified but study relating to four hospital admissions	n/a	Personal diaries and medical documents including community and hospital clinical records relating to four long term (4–8 month) inpatient admissions	Autoethnography

IP, inpatient; SSI, semi-structured interviews; DA, discourse analysis; IPA, interpretative phenomenological analysis; TA, thematic analysis

### Quality assessment and risk of bias

The evaluation of the studies found variable quality across the articles (see Additional file 1: Table A). The authors note that research design and rationale was unclear in five studies, and the consideration of the relationship between authors and participants was insufficient in three studies. Additionally, there was a paucity of information regarding in-patient treatment protocol, patient admission and eating disorder duration. Furthermore, there was variability in quality of rigour in data analyses, with approximately half of the studies producing more descriptive or superficial analysis while others were more in-depth.

Potential biases across studies included: inconsistency in the time-point of data collection (during, immediately post treatment or retrospectively); lack of consideration for key factors, such as illness severity/duration, length of admission and a number of therapies; lack of inclusion of interview questions to assess for potential biases in the collection of data and the interviews’ direction; and an over-reliance on single coders/data analysts and under-reliance on participant member checking. Within the studies, samples were not representative, with minimal male participants and a lack of cultural diversity (including Indigenous peoples).

### Thematic findings

**Fig. 2:**
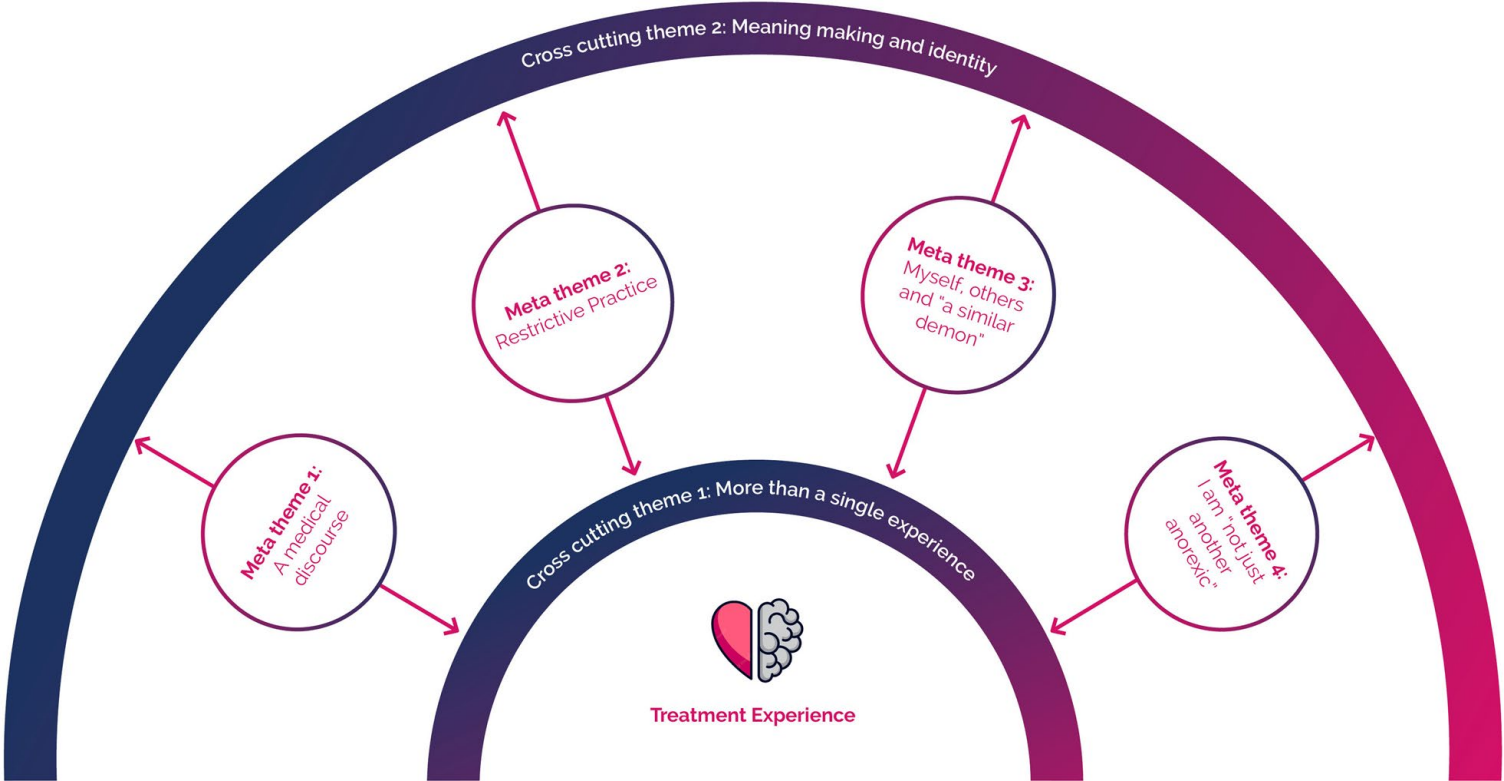
Relationships between meta-themes and cross-cutting themes observed in patient lived experiences of inpatient treatment for anorexia nervosa

Four meta-themes were constructed from the data: (1) a medical discourse—“I don’t think it’s individualised here”; (2) restrictive practice—living in a “bubble”; (3) myself, others and “a similar demon”; and (4) I am not “just another anorexic”. Two themes cut across the data: (1) more than a single experience; and (2) meaning making and identity. As depicted in Fig. 2, the four meta-themes connect through two cross-cutting themes. For example, participants’ positioning on the dominant medical discourse had implications on their meaning making throughout the treatment experience. As noted above, only previously published data are presented in these findings. Additional extracts from participants and from the authors of primary studies for each theme can be found in Additional file 3: Table B).

### Meta-theme 1: a medical discourse—“I don’t think it’s individualised here”

On admission to an inpatient facility for the treatment of AN, a patient’s family/carers and members of their treating teams will have legitimate concerns about an individual’s weight loss and the associated health complications [3, 48]. As such, there is a strong focus on addressing physiological symptoms of an eating disorder (e.g., medical stabilisation, nutritional rehabilitation and weight restoration) through standardised phase-based treatment protocols [49]. Within this context, participant accounts of their inpatient treatment experiences highlighted challenges presented by the dominant medical discourse and the systemic focus on addressing physiological symptoms of an eating disorder [46, 47, 50].

Participants across seven studies [38, 39, 41, 42, 44, 46, 47] expressed disappointment and, at times, exasperation with what they felt was an almost exclusive focus on physiological rehabilitation at the perceived expense of their psychological wellbeing, individual identity, personal values and treatment goals. Colton and Pistrang [38] noted in their analysis that participants felt the main aim of the inpatient treatment unit was to “fatten them up” and restore weight, rather than support psychological recovery and wellbeing. This interpretation was supported by participants in Kezelman and colleagues’ study: “… essentially this place isn’t about getting better… psychologically, just… physically…” [42], P8]

Malson and colleagues suggested that the medical discourse, while necessary in the inpatient treatment setting, created an environment where “the eating disordered patient” may be constructed as being “entirely pathological” [37], p483]:

And they don’t listen to you at all. And whenever you try and like rationalize anything with them they just, you get told to sort of shut up because it’s the illness talking and you can’t possibly know any better than them because otherwise you wouldn’t be in here in the first place. [37] (Jessica M)

Similarly, in narrating her experience of multiple inpatient admissions, O’Connell wrote:

In treatment, anorexia was reductively constructed as a pathologised, medicalised condition, and while in some ways affirming, this also sometimes led me to feel misunderstood, invalidated and stereotyped. Anorexia became the overriding source of my identification, leading to my behaviour being automatically interpreted as symptomatic of illness, resulting in me feeling powerless. In addition, being unable to make my own decisions felt child-like and I found this humiliating. [47], p247–245]

Within the dominant medical discourse, the staff are in the position of being the expert authority (e.g., “you can’t possibly know any better… because otherwise you wouldn’t be in here in the first place.” [37] (Jessica M)). Thus, the patient was constituted as “powerless” [47] within their own treatment journey, as there was little or no place for them to be an “expert” in understanding their own symptoms through the lens of their lived experiences.

I don’t think it’s individualised in here…. They have their formula and they just put everyone on it… [but] everyone’s problems here are completely different…. [42], p10]

Participants’ experience of being “othered” [47] within the dominant medical discourse was most apparent in the frequent expressions of frustration regarding the perceived lack of individualisation in treatment, particularly during the initial phases of treatment [37, 39, 41, 43, 44, 46, 47]. Where participants felt “othered” during their treatment, they reported questioning treatment efficacy and goodness of fit—“is the right sort of treatment for me…” [37] (Clare)—and reported engaging in acts of resistance in an attempt to preserve their identity in the face of a dominant medical discourse:

… even if they get a guardianship order, what are they going to do? Stand around and psychoanalyse you against your will…. It’s only physical things that they can only do to me really. [39] (Jessica B)

### Meta-theme 2: restrictive practice—living in a “bubble”

Participants across all the studies described the inpatient treatment setting as being one of highly regimented schedules and practices that spatially, relationally and temporally separated them from external experiences. As highlighted by Eli [41] and others [42, 44–47], the majority of participants regarded the restrictive treatment environment with a sense of ambivalence. While many participants expressed a strong dislike of the restrictive treatment environment [37, 39, 41, 43, 44, 46]—“It’s a place for hell… you’re stuck here and you can’t get out and you can’t do anything” [38] P17]—they also perceived the same environment as being instrumental in creating a “safe space” or “safety bubble” [43], P4] that separated them from the “real world” [47] or “outside world” [41]: “It kind of became your safe haven.”.[44], P6]

Many participants reflected that the restrictive treatment environment allowed them to engage in treatment by removing opportunities to engage in unhelpful behaviours associated with the eating disorder [38, 41, 43, 44, 46, 47]. For example:

It [the ward] was a little lab like that, that you could be inside…. A lab in the sense that it was very sterile, it was – very very exact and measured conditions, and – you knew that you, it’s not like the real world, so it eased [our burden]. [41] (Grace)

You don’t have to control it [the eating disorder] anymore and you can give over that control… it feels as if you are in that stage where you can’t make any decisions… so it is nice to have other people take over. [43] (Participant 17)

As exemplified in the extracts above, the treatment environment—with its clear daily routines, activities and dietary programs—provided participants with a clear anchor and structure within which to relinquish control of the eating disorder [41–44]. The physical separation from their external world also provided some participants with a space for self-discovery and growth [41, 43, 47] Meital explained:

The first hospitalization (laughs) – its funny to say but I enjoyed it. Like, suddenly I had friends, and it was really pleasant, and it was also, somehow, [a way of] getting out of home, something that I wanted. I wanted my privacy and my independence, and I had it there. [41]

While many participants expressed their dislike of the inpatient treatment environment, several studies [38, 41, 42, 44, 46, 47] highlighted participants’ apparent reluctance or apprehension to leave treatment and the perceived safety of the structured/boundaried treatment environment. For example, one participant in Smith and colleagues’ study stated, “You become dependent on it… you feel it is your safe place almost. I am almost afraid to be here now because I have become quite attached.” [43] (Participant 6) Tali, meanwhile, stated, “I didn’t want to leave, I didn’t want to leave, no one wanted to leave… as difficult as it was, there were many difficult things, but—but it was sort of a greenhouse” [41].

The transition away from high-intensity, wrap-around supports back into the community was experienced as a source of fear for many participants—“I didn’t want to leave” [41] (Tali)—with participants citing a perceived lack of support in the community [43, 47]. Other participants questioned their ability to take a stand against their eating disorder alone outside of the ward: “I worry about going home full time… I hear this voice saying… it will be you and me again.”.[43] (Participant 3)

### Meta-theme 3: myself, others and “a similar demon”

A unique aspect of inpatient treatment for AN is living in an environment with others experiencing the same condition. For many participants, admission to an inpatient facility is the first time they had met or interacted with other individuals with an eating disorder [38, 41, 44]. Participants across six studies described fellow patients as having a major impact—for better and for worse—on their inpatient experience [37, 38, 41–44]. For many participants, the inpatient community functioned as a formative experience that was central to the narrative of their inpatient treatment experience. As Alon explained:

… getting there, and sitting in groups, and hearing people talking about things that you’re also going through – there’s something very powerful in this, in this sense of ‘I’m not alone’… [we] feel like, we’re all dealing here with a similar demon, and there’s some sense of shared destiny. [41]

Participants in six studies [37, 38, 41, 43, 44, 47] spoke of the importance of identification with other patients in reducing isolation, as well as normalising and validating their affective experience:

… you can talk to them [patients] so much easier than what you can when you’re out of here. Do you know what I mean, you can talk about anorexia just as you can talk about Coronation Street [a television show] … whereas at home, that issue would be totally avoided and I would not even talk about it. [38], P10]

Participants also spoke of learning effective coping skills from their peers, as well as finding hope for recovery: “It is really good in terms of being able to hear how other people have gotten over the drive to exercise and how they have managed to eat certain foods.”.[43] (Participant 3)

Despite the positive aspects of being part of an inpatient community, participants in seven studies [38, 41–44, 46, 47] spoke of making physical and behavioural comparisons with others: “I saw other people that were thinner than me and it made me feel like I had failed at my eating disorder.” [44], P2] Participants described experiencing feelings of “envy” towards their “emaciated peers” [43], p23] and competing to be “the best anorexic” [38], P8]. Over half of participants in Eli’s [41] study reported feeling “triggered” by the close proximity of other patients and being able to “observe” other patients’ appearance, progress and “everyday practices”, with one participant explaining, “Since we’re all eating in the same room, you’re experiencing everyone else’s troubles.”.[44], P6]

The presence of other patients at different recovery stages also appeared to, at times, exacerbate the distress associated with between-patient comparison. As the following participants explained:

When you reach a condition that’s relatively healthy and fine and you’re halfway there… suddenly a girl who weighs 20 kilos shows up… I don’t want to see it…. It’s not that it’s the sick side [of me], it’s like – it’s the side I never had. So why do I need to get acquainted with it? [41] (Natalie)

It really screws me up seeing extremely thin people [. . .] they are pleased they are not as fat as I am. [47] (Diary, 18th January 2007)

Furthermore, several participants described a contagion effect among the inpatient community. Participants reflected that living with other patients made them more aware of and susceptible to adopting the unhelpful behaviours of others. For example, one young person stated: “I didn’t really know … about self-harm, um, about pacing to stop your weight going up, you know, walking around, exercise. I soon cottoned on.” [38], p311] Another said, “Seeing what they [other patients] did kind of gives you ideas about being sneaky.”.[44], P1]

### Meta-theme 4: I am “not just another anorexic”

Participants repeatedly emphasised the importance of healthcare professionals and treating teams seeing them as individuals. Colton and Pistrang [38] noted that a “key dimension” (p310) used by participants in describing their experiences of inpatient treatment was whether they felt staff viewed them as being an individual or “just another anorexic” (p310) coming through the program. For example:

I miss just being me, not a patient in need of help and support. [45], p5]

It’s sort of like speaking to him [a doctor] is like bashing your head up against a wall… Because everything you say is part of the disease. No matter what it is… And you’re like: I’m a person. There’s a personality in here you know?… You know I’m not just anorexic. [37] (T6A—interviewer responses removed)

Across several studies, participants perceived staff as being too busy and not having enough time to listen to them or care about what they did, provided they complied with treatment [38, 39, 41–43, 47] Participants frequently expressed their frustration at the perceived lack of individualisation in their treatment planning and often reported feeling “pigeonholed” [37] (Polly) by clinical staff (e.g., “I also frequently felt ‘unheard’ and my reasoning invalidated due to my inability to escape an anorexic framing” [47], p274]), particularly during assessment and the initial phases of treatment [39, 41–43].

Participants’ feelings of being misunderstood by clinical staff appeared to foster a climate of resistance within the inpatient treatment setting [39, 41, 44]: “it makes you not want to cooperate because they don’t really want to understand.” [42] Conversely, “good staff” [43–45] were described as able to “… see the person behind the anorexia.” [38], P5] As Grace reflected:

They knew about me much more than I knew about myself… things that even I wasn’t aware of, but that they could see from the outside.... It always gave me a good feeling – that I don’t have to talk and they still know. [41]

Where participants felt seen and acknowledged as an individual—not merely the bearer of an eating disorder diagnosis—they reported increased engagement in recovery-orientated/help-seeking behaviours. For example:

When they’re more encouraging and supportive it makes me want to try harder and when they’re more forceful it makes me always want to pull against and try harder at doing the wrong things. [38] (Participant 9)

… you build up trust… you know you can say things to them and they understand a bit more because they know more about your past. [43] (Participant 2)

Boughtwood and Halse suggested that “recognizing the differences between individual patients and respecting the meanings they attach to their illness is central to the therapeutic alliance in the treatment of anorexia.” [39], p92] Furthermore, the ability of staff to “hold hope” [23] for a patient’s recovery appeared to strengthen participant motivation and connection to a sense of self or identity beyond their eating disorder [38, 39, 41, 44]. As a participant in Kezelman and colleagues’ study recalled, “Wow, these people… [have] faith in me, I need to have faith in myself.”.[42], P8]

### Cross-cutting theme 1: more than single experience

Inpatient treatment for AN typically entails several weeks or months of living away from home. In reflecting on treatment experiences, participants across all studies outlined a multidimensional experience characterised by a series of non-liner—often recursive—phases or transitions. Kezelman and colleagues [42] observed three broad phases in their analysis of the adolescent inpatient treatment experience: (1) reconciling with the AN diagnosis and understanding the necessity of medical intervention; (2) adjustment to treatment and the treatment environment; and (3) reflection and integration. They noted that participant accounts of treatment experience demonstrated a “complex and often ambivalent psychological process” whereby individuals’ understandings and “acceptance of their physical and medical needs were often in conflict” (p228) with their “affective experiences” and beliefs regarding recovery.

The concept of transitions during treatment were exemplified throughout O’Connell’s [47] autoethnographic account of her inpatient treatment experience across multiple admissions, and further highlighted by Smith and colleagues in their theme “Experience of transition” [43], p21]. They noted participants’ initial struggle to adjust to the treatment environment—“at the start, I didn’t want to be here” (p21)—before coming to see “treatment as a safe environment” (p21) they felt reliant on prior to discharge. Patients’ experience of transitioning between phases during treatment for AN were directly and indirectly observed across all studies in this synthesis. As demonstrated in meta-themes 1 to 4, participants’ experiences appeared to be influenced by how they navigated and made meaning of these transitions during treatment.

### Cross-cutting theme 2: meaning making and identity

Participant accounts of inpatient treatment experience across all studies were characterised by conflicts and dilemmas, or the experiences of ambivalence and liminality throughout treatment. Although participants varied in their views regarding the helpfulness of inpatient treatment, most individuals reported a sense of duality—both positive and negative feelings—regarding multiple aspects of the inpatient treatment experience (meta-theme 1 to 4). At times, participants appeared overwhelmed by internal conflicts regarding their diagnosis and the necessity of inpatient treatment (meta-theme 1 and 2), their experience of staff (meta-theme 1 to 3) and other patients (meta-theme 4), and the restrictive treatment environment (meta-theme 2 to 4).

Participants’ ability to make meaning of their experiences and resolve ambivalence at various phases throughout the inpatient treatment journey appeared to shape their global perception(s) of the inpatient treatment. For example, as outlined in meta-theme 2, Meita [41] described her first inpatient experience as being a positive experience that provided her with independence. However, she described her second inpatient experience extremely differently:

The second hospitalization, in comparison, was very traumatic. I felt really bad there. I couldn’t find myself…. Being in a closed ward with very tough discipline, very clear rules, where they decide for you when you’ll eat, when you’ll have time for breaks, like – it didn’t suit me anymore. I needed my freedom, to decide on my own structure.(p7)

In analysing this shift, Eli [41] noted that while the “ward itself had remained the same”, Meital’s desire for “freedom”, likely her definition of what independence looked like, and priority to “find myself” (p7) had shifted between admissions, thus leading her to experience and engage with the same treatment facility and protocol in two very different ways.

Participant meaning making also appeared to be influenced by individual readiness for change. A central conflict described by many participants was whether or not they were willing to “let go” of the eating disorder and participate collaboratively in treatment. For example, O’Connell reflected that in “wanting something different, I tentatively opened up in my mind to the idea of letting go of anorexia.” [47], p275]. While participants consistently identified that their own willingness for recovery was central to treatment success—“I have to wait ‘till I am ready” [38], P6]—Broughtwood and Halse [39] observed that some patients managed this conflict by temporarily performing the role of the “perfect (obedient) patient” (p89), as yielding to the clinical team at times served their longer-term personal agenda.

There is an ‘us versus them’ mentality though, like [the doctors] want me to put on [a certain amount of weight] by Wednesday and um I can’t believe it, and you know. Yeah it’s hard to explain but, there is a real ‘I’ll do it [gain weight] just to make them happy so that I can get home’. Ah, rather than ‘they think that it’s best that I put on this amount of weight, and they know what they’re doing because they’re medical professionals, so I guess it is best for me.(Renee)

Renee’s engagement with the medical discourse was more complex than simply “obeying or rejecting” (p880) her clinical team or treatment; rather, her position in this discourse was one of ambivalence regarding whether the treatment goals proposed by her treating team were of benefit to her. Renee’s positioning in relation to the dominant medical discourse appeared to impact her motivation and the way in which she interpreted clinical staff actions and the restrictive treatment environment.

Participants’ values, treatment goals and connection to an identity outside of their eating disorder identity also appeared to have an impact on how participants made meaning of their eating disorder diagnosis and treatment experiences. Throughout treatment narratives [37, 40, 41, 43–47], AN was experienced by participants as being both a “friend” and identity investment*—*“a shield to hide behind, and something which gave confidence and security”—as well as being an “enemy” or a “suffocating, frightening and depriving” [38], p310] identity thief (see Additional file 3: Table B for exemplar quotes). Thus, treatment engagement and recovery for participants was not simply a process of choosing to disengage from a set of unhelpful behaviours associated with their eating disorder, but rather the acknowledgment of AN and the consideration one’s own identity, values and purpose outside of the eating disorder [38, 41, 43, 44, 46, 47]. As one participant explained:

I find it difficult to distinguish… what is me and what is the eating disorder… a lot of what my treatment has been is actually finding my own identity. [43] (Participant 3)

## Discussion

This meta-analysis sought to synthesise contemporary literature pertaining to individuals’ lived experiences of residential and inpatient treatment for AN within eating disorder-specific treatment services. Eleven qualitative studies were selected with a total of 159 participants with lived experience of inpatient treatment for AN. Four meta-themes emerged from the data: (1) a medical discourse—“I don’t think it’s individualised here”; (2) living in a “bubble”; (3) myself, others and “a similar demon”; and (4) I am more than “just another anorexic”. The data also revealed two cross-cutting themes: (1) more than a single experience; and (2) meaning-making. These themes highlight the complex and multifaceted nature of inpatient treatment experiences.

Findings from this synthesis suggest that, while many individuals retrospectively acknowledge the necessity of medical intervention as part of their treatment journey, the restrictive treatment environment and biomedical focus of inpatient treatment facilities often disqualifies the patient’s voice, individual identity, lived experience, personal values and understandings of their symptoms. These findings are consistent with the broader body of literature pertaining to inpatient experiences of hospitalisation for psychiatric care, which highlight patients’ sense of feeling restricted or trapped in a different world during admission [26, 51–53]. Within the dominant medical discourse, clinical staff are frequently positioned as being expert authorities and may be perceived as “prison wardens”, thus leaving little or no space for patients to be an “expert” in understanding their own symptoms through the lens of their lived experiences [25, 53–55].

Inpatient admissions for psychiatric care represent a significant disruption to an individual’s life narrative, sense of self and identity [25, 52]. As such, inpatient treatment may be one of the “most challenging experiences” [51], p329] over the course of an individual’s illness and recovery journey. Participant accounts of inpatient treatment of AN in this synthesis were characterised by the experiences of ambivalence and liminality. While many individuals expressed a desire for recovery and a life beyond AN, the concept of recovery was closely associated with complex identity negotiations and hindered by a fear of the unknown in recovery [23, 56–59]. These findings speak to the ego-syntonic nature of AN (e.g., the way in which AN behaviours may align with an individual’s ideal self, values and identity) [57, 60] and highlight the paradoxical way in which those with AN may simultaneously wish for recovery while actively resisting treatment [61].

As inpatient treatment for AN typically occurs at critical points in an individual’s treatment journey, participant treatment experiences may reflect their broader experience of liminality in their relationship with themselves and AN. The concept of recovery was associated with complex identity negotiations across participant narratives in this study. Thus, treatment engagement was not simply a process of an individual choosing to disengage from a set of unhelpful behaviours associated with an eating disorder, but rather the acknowledgment of AN and the consideration of one’s own identity, values and purpose outside of the eating disorder. This is consistent with research indicating that recovery from AN is more about the reclamation of self and identity outside of AN than it is the illness process [25, 58, 62, 63]. The way in which patients made meaning of their experiences and resolved ambivalence throughout their inpatient treatment journey shaped their global perception(s) of the inpatient treatment. As such, factors independent of treatment (e.g., life events, personal values, identity, self-reflection, life goals, personal understandings of AN) are likely to influence individual motivation for change [56, 59, 64].

Participant narratives across all studies highlighted the inherent conflicts between service providers administration of standardised phase-based treatment protocols, the broader therapeutic milieu and patients’ desire for person-centred care. For example, many participants felt the inpatient treatment programs focused too heavily on the physiological symptoms of AN and provided a lack of assistance in addressing the underlying psychological difficulties and the distress associated with weight gain. This finding is consistent with current literature [25] and adds weight to outcome studies suggesting the presence of a gap between physiological (e.g., weight) and psychological (e.g., eating disorder cognitions, level of distress relating to weight and shape) improvements following inpatient treatment for AN [65, 66]. Evidence suggests without improvements in both physiological and psychological aspects of the AN, there exists a risk of a pseudo-recovery—that is, a physical recovery in the absence of psychological recovery—which may place an individual at higher risk of relapse following discharge [67, 68]. Thus, while nutrition rehabilitation is essential in ameliorating both psychological and psychosocial symptoms of AN [49, 68, 69], individual aspects such as motivation for change are also important to consider in predicting treatment adherence and patient outcomes [22, 70].

Clinical staff were found to play a critical role in the formation of patient treatment experiences and in the creation of the ward milieu. As with previous literature [25, 53, 61, 71], where patients feel seen as an individual—“more than just anorexic”—they were more likely to engage in recovery-orientated behaviours [65, 70]. Furthermore, the ability of staff to “hold hope” [23] for a patient’s recovery appeared to strengthen participant motivation and connection to a sense of self or identity beyond their eating disorder. Similarly, the inpatient community was seen as a formative experience—for better or for worse—for many participants and was central to their narrative of their inpatient treatment experience. While patient peers served to normalise and validate the inpatient treatment experiences, several participants described a contagion effect among the inpatient community.

### Clinical implications

The tensions between administering replicable standardised phase-based treatment protocols and patients’ desire for person-centred care are many (e.g., the conflict between the necessity of medical interventions and treatment non-negotiables vs the development of positive therapeutic relationships and maintenance of a positive patient milieu) and may pose the greatest challenge for healthcare professionals in treating AN in the inpatient setting [25, 53, 68, 72]. Participant narratives of inpatient treatment indicate that addressing these conflicts is of the utmost importance. Explicitly acknowledging conflicts—where clinically relevant—may allow healthcare providers to provide patients with appropriate and timely information regarding treatment decisions. For example, the provision of a clear and individualised treatment rationale—that includes the persons’ own goals along with treatment non-negotiables [73]—maximises client autonomy in the face of a dominant medical discourse [26, 70, 71, 74, 75].

Eliciting patients’ own treatment goals and understandings of the function(s) of their illness early in treatment may create opportunities for the exploration of non-treatment related factors associated with the tipping point of change (e.g., values, relationships and individual identity separate from AN) later in treatment. It may also facilitate the development co-ownership in patient treatment journeys, particularly where patients are unable to provide consent due to involuntary admission [26, 46, 76]. Furthermore, adopting a person-centred treatment approaches, which keep the individual with their unique experiences at the core of treatment planning and assessment, may enhance the effectiveness of tailored inpatient treatments for sub-populations of those presenting with AN (e.g., ethnic minorities, individuals with a trauma history or a co-occurring diagnosis such as autism) [74, 77–79]. Treatment context may also have influenced participant experiences; for example, inpatient treatments may be more prohibitive and focus more on eating behaviour change/symptom management to mitigate medical risk.

Clinical practice guidelines for the treatment of eating disorders [49], suggest that treatment for those with AN be provided within a treatment framework that “supports the values of recovery-oriented care”. (p6) This approach recognises that no two individuals are the same and recognises the inherent strengths and capacity each individual holds within themselves. As such, recovery-orientated approaches to mental health treatment aim to promote self-direction, self-determination, self-management and autonomy, in the context of individualised, holistic and evidenced based person-centred treatment.

Adopting person-centred and recovery-orientated treatment approaches that prioritise patient safety and autonomy needs to be balanced with safety and broader/stakeholder considerations (e.g., the ability to operationalise quality interventions in a replicable way). Findings in this review support the view that inpatient treatment may be more efficacious when focused on both the physiological and psychological symptoms of AN. Building patients’ ability to cope with and tolerate distress associated with weight gain during inpatient treatment may assist in closing the gap [65, 66] between physiological and psychological improvements following inpatient treatment, thus reducing participant risk of relapse following discharge [67, 68].

### Strengths and limitations

The findings from this study need to be interpreted in the context of several limitations. First, given the exclusion criterion, the authors may have excluded pertinent research published in grey literature or languages other than English. Second, the authors only included previously published data in this meta-synthesis. As such, a significant proportion of original transcript data were not synthesised in this study. Third, the themes generated in this paper are influenced and shaped by the authors’ focus on participant lived experiences.

A strength of this study was its exploration of a combined 159 participants’ lived experience of inpatient treatment for AN from 11 separate studies. Overall, the included studies were of good quality. However, the authors note that descriptions of participant demographics (e.g., age, ethnicity, socio-economic status, severity of illness and treatment history) were limited. Similarly, the descriptions of treatment programs and settings—including the structure, delivery and content of treatment modalities—were limited. The authors also noted an underrepresentation of male participants and the absence of qualitative literature regarding patient lived experiences of residential care. The lack of consistent descriptive data limited the authors’ ability to assess patient and treatment variables that contribute to patient lived experiences and limits the generalisability of the findings. Furthermore, variability in sampling and recruitment strategies used in the included studies may have led to the possibility of selection bias and skewed views of treatment. Further research is also needed to determine if there are differences in patient experiences across the lifespan and, if so, how interventions may be best tailored to meet the needs of patients in different life phases (e.g., young persons and adults).

Despite these limitations, this synthesis has a number of strengths, including being a response to the paucity of research in relation to the lived experience of inpatient treatment for AN. The authors also employed a rigorous methodological process in the selection, evaluation and interpretation of the studies in this synthesis. This included several authors working in parallel on steps in the interpretation and analysis of data drawn from a number of studies. By drawing on the voice of those with a lived experience of inpatient treatment for AN within eating disorder-specific treatment services, this study may generate a map for healthcare professionals as they navigate the inherent conflicts between administering standardised phase-based treatment protocols and patients’ desire for person-centred care.

## Conclusions

Results of this synthesis suggest that the lived experience of inpatient treatment for AN within eating disorder-specific treatment services is complex and multifaceted. Inpatient treatment for AN typically occurs at critical points through a patient’s treatment journey and represent a significant disruption to an individual’s life narrative, sense of self and identity. As such, patient narratives are marked by conflicts and reflect participants’ sense of liminality in their relationship with themselves and AN. This supports research indicating that recovery from AN is more about the reclamation of self and identity outside of AN than it is the illness process [25, 59, 64, 73].

While many individuals retrospectively acknowledge the necessity of medical intervention as part of their treatment journey, the restrictive treatment environment and biomedical focus of inpatient treatment facilities often disqualifies the patient’s voice, individual identity, lived experience, personal values and understandings of their symptoms. Furthermore, without improvements in both physiological and psychological aspects of the AN, there exists a risk of a pseudo-recovery, which may place an individual at higher risk of relapse following discharge. Adopting person-centred and recovery-oriented treatment approaches may serve to maximise client autonomy in the face of a dominant medical discourse and support patient reclamation of identity. However, further research is needed to identify how service providers may best navigate the inherent conflicts in balancing the necessity of medical and psychological intervention within phase-based treatment protocols with person-centred treatment approaches in the treatment of AN.

## Supplementary Information

**Additional file 1.** Table A: Quality assessment of papers included in review.

**Additional file 2.**  Researcher position statements.

**Additional file 3.** Table B: Exemplar data extracts for metathemes in the metasynthesis.

## Data Availability

The additional files document contains the following data and materials for the meta-synthesis: Assessment of quality of included papers (Additional file 1: Table A), researcher position statements (Additional file 2) and Exemplar data extracts for Metathemes in the Metasynthesis (Additional file 3: Table B). No additional data is available.

## Abbreviations

AN: Anorexia nervosa
DSM-5: Diagnostic and Statistical Manual of Mental Disorders (5th Edition)
ICD-10: World Health Organisation International Statistical Classification of Diseases and Related Health Problems
