# Supplementary material for: A systematic review of people’s lived experiences of inpatient treatment for anorexia nervosa: living in a “bubble”
Source: J Eat Disord. 2023 Jun 9;11:95. doi: 10.1186/s40337-023-00820-0 (PMC10257311; doi:10.1186/s40337-023-00820-0)
Supplement: Supplementary file 1 — Additional file 1. Table A: Quality assessment of papers included in review. [file 40337_2023_820_MOESM1_ESM.docx]

| **Additional File 1** | | | | | | | | | | | | | | | | | |
| --- | --- | --- | --- | --- | --- | --- | --- | --- | --- | --- | --- | --- | --- | --- | --- | --- | --- |
| **Table A.** Quality assessment of papers included in review | | | | | | | | | | | | | | | | | |
|  | **Quality Criteria** | | | | | | | | | |  | | |  | | |  |
| **Author(s) (year)** | **(1)** | **(2)** | **(3)** | **(4)** | **(5)** | **(6)** | **(7)** | **(8)** | **(9)** | **(10)** | | **Average** | **Overall rating** | |  |  |  |
| Malson et al., (2004)^37^ | 3 | 3 | 3 | 3 | 3 | 3 | 3 | 2 | 3 | 3 | | 2.9 | ++ | |  |  |  |
| Colton & Pistrang (2004)^38^ | 3 | 3 | 3 | 3 | 3 | 3 | 3 | 3 | 3 | 3 | | 3 | ++ | |  |  |  |
| Boughtwood & Halse (2010)^39^ | 3 | 3 | 3 | 3 | 3 | 2 | 3 | 3 | 3 | 3 | | 2.9 | ++ | |  |  |  |
| Long et al., (2011)^40^ | 3 | 3 | 1 | 3 | 3 | 3 | 3 | 3 | 3 | 3 | | 2.8 | ++ | |  |  |  |
| Eli (2014)^41^ | 3 | 3 | 3 | 3 | 3 | 3 | 3 | 3 | 3 | 3 | | 3 | ++ | |  |  |  |
| Kezelman et al., (2016)^42^ | 3 | 3 | 1 | 3 | 2 | 1 | 3 | 3 | 3 | 3 | | 2.5 | ++ | |  |  |  |
| Smith et al., (2016)^43^ | 3 | 3 | 1 | 3 | 3 | 3 | 3 | 3 | 3 | 3 | | 2.8 | ++ | |  |  |  |
| Thabrew et al., (2020)^44^ | 3 | 3 | 1 | 3 | 3 | 3 | 3 | 3 | 3 | 3 | | 2.8 | ++ | |  |  |  |
| Solhaug & Alsaker (2021)^45^ | 3 | 3 | 1 | 1 | 1 | 1 | 2 | 1 | 3 | 3 | | 1.9 | + | |  |  |  |
| MacDonald et al., (2023)^46^ | 3 | 3 | 1 | 3 | 3 | 3 | 3 | 3 | 3 | 3 | | 2.8 | ++ | |  |  |  |
| O’Connell (2023)^47^ | 3 | 3 | 3 | 3 | 3 | 3 | 3 | 1 | 3 | 3 | | 2.8 | ++ | |  |  |  |
| **Mean score for each criterion** | 3.0 | 3.0 | 1.9 | 2.8 | 2.7 | 2.5 | 2.9 | 2.5 | 3.0 | 3.0 | | 3.0 |  | |  |  |  |
| (1) Clear statement of aims/objectives of the research.  (2) Appropriateness of qualitative methodology.  (3) Research design is clear and appropriate for the research aims.  (4) Recruitment strategy appropriate to the aims of the research.  (5) Data collection appropriate to research issue.  (6) Consideration of relationship between researcher and participants (e.g. reflexivity, critical examination of researcher’s own role, personal biases etc.).  (7) Ethical considerations (approval sought, informed consent, confidentiality, effects of the study on participants etc.).  (8) Rigor in data analysis (analysis clearly described, consideration of persons conducting analysis etc.).  (9) Clear statement of findings (findings clearly supported by the data and/or credibility of findings discussed).  (10) Contribution and implications to existing knowledge of the research, while outlining its limitations and future directions.  3 = Yes well addressed; 2 = Can’t tell if this was addressed; 1 = No not addressed. | | | | | | | | | | | | | | | |  |  |
