# Supplementary material for: A systematic review of people’s lived experiences of inpatient treatment for anorexia nervosa: living in a “bubble”
Source: J Eat Disord. 2023 Jun 9;11:95. doi: 10.1186/s40337-023-00820-0 (PMC10257311; doi:10.1186/s40337-023-00820-0)
Supplement: Supplementary file 2 — Additional file 2. Researcher position statements. [file 40337_2023_820_MOESM2_ESM.docx]

# Additional File 2: Researcher positioning statements

**Rebekah Rankin:** I am a Anglo-European female, Clinical Psychology Registrar and PhD candidate, with a lived experience of disordered eating. My research and clinical work is primarily in the field of eating disorders. To ensure analytical quality and rigour in the present study I kept a self-reflective journal (Nowell et al., 2017) during all stages of data transcription and analysis. I also attended fortnightly supervision sessions (with JC, LR, PH) to capture/process my personal and professional responses to the research process and findings to reduce their influence on data analysis.

**Janet Conti:** I am a Clinical Psychologist, Dietitian and academic in Clinical Psychology. My research and clinical work seeks to prioritise the voice of the experiencing person to inform the development of a greater number of effective treatment interventions for AN that are tailored to the needs and preferences of the experiencing person and their family.

**Lucie Ramjan:** I am a Registered Nurse and Professor of Nursing. My research expertise is qualitative and has centred on learning more about the personal experiences of treatment and recovery for people with eating disorders. This research supports finding adjuncts to treatment that empower and inspire hope for recovery.

**Phillipa Hay:** I am an academic Psychiatrist with long-standing clinical and research experience in the treatment of people with anorexia nervosa. I am very interested to explore and understand better how to improve treatments, reduce distress during treatment, and in particular to better understand why some people have poor outcomes.
