# Supplementary material for: A systematic review of people’s lived experiences of inpatient treatment for anorexia nervosa: living in a “bubble”
Source: J Eat Disord. 2023 Jun 9;11:95. doi: 10.1186/s40337-023-00820-0 (PMC10257311; doi:10.1186/s40337-023-00820-0)
Supplement: Supplementary file 3 — Additional file 3. Table B: Exemplar data extracts for metathemes in the metasynthesis. [file 40337_2023_820_MOESM3_ESM.docx]

| **Additional File 3** | | |  |
| --- | --- | --- | --- |
| **Table B.** Additional extracts | | |  |
| **Meta theme 1: A medical discourse—“I don’t think it’s individualised here”** | | | |
| **Source** | | **Extracts** |  |
| Malson et al.,^37^ | | - Julie: Everything I say he [a doctor] just like, he laughs at you kind of thing. Like you say one thing and he just laughs and he goes: Oh it’s not her talking, it’s the illness. (T7A) - Jessica: And they don’t listen to you at all. And whenever you try and like rationalize anything with them they just, you get told to sort of shut up because it’s the illness talking and you can’t possibly know any better than them because otherwise you wouldn’t be in here in the first place. / Int: mm/ [sighs] And there’s like no compromise or anything. You’re not allowed to have like choices or preferences. (T14UK) - Elizabeth: I also think talking down to us, like everything is your anorexia and having everything you do analysed as anorexic pattern is very destructive. / Int: mm / I know that they are desperate to find why people are ill and to challenge everything because of that, but there are ways of doing it. (T13UK) - “I question whether this is the right sort of treatment for me because I feel that my problems aren’t addressed at all and I’m just um (.) being treated as a case book, study sort of thing, out of a textbook.” (Clare) - Polly: I thought he [a psychiatrist] was pigeon-holing me. / Int: right, as an anorexic? /Yeah, yeah. And all anorexics think this and do this / Int: right/so you must be like that . . . I didn’t trust any of his advice / Int: right / because I felt he was telling me about what, um, you know, your classic anorexic ought to do. / Int: mm/ And I always felt well he doesn’t know me. So / Int: right / he’s just seeing the illness part of me. He’s not taking me as a whole. (T8UK) |  |
| Colton & Pistrang^38^ | | - ‘It just feels like the physical perspective is a little too . . . out of perspective in comparison to the mental, because to me the mental problems are what have caused physical problems and it doesn’t seem to be working that way in terms of - ‘I just wish they would have helped me with the fact that I needed help with calming myself down, rather than just increasing my diet which made me panic.’ (P13) - ‘I feel that people need therapy on a more day to day basis, you know it doesn’t have to be as deep and as long as, as those sessions but, you just, you can’t switch on and off.’ (P3) - It just feels like the physical perspective is a little too . . . out of perspective in comparison to the mental, because to me the mental problems are what have caused physical problems and it doesn’t seem to be working that way in terms of recovery. (P3) |  |
| Boughtwood & Halse^39^ | | - I saw it as more officially a problem once it was recognized by Dr F, you know, like medically. He told me you have to do something about it. . . because before that it just seemed like oh, if I just ignore it, it’ll go away. But until then until he actually said you know, you do have a problem, there’s something you have to do, it sort of made it, made it more concrete and that it was a big problem. (Renee) - Rita comment on medical monitoring: “You don’t feel much different when it’s that low. Like they say you could go into cardiac arrest or whatever, but it doesn’t feel like that, you just feel normal. (Rita) |  |
| Long et al.,^40^ | | - For Participant 1 mealtimes were “ … like playing a game”, and went on to explain: “ … because it seems so rehearsed and it doesn’t seem individual … It just leaves me cold”. - Participant 5: “They [staff] probably know from reading about it, but they don’t know from experience. So it’s not like they know how we deal with it”. |  |
| Eli^41^ | | - When I was at [the ward] and I had a [GI condition] and it was really painful, and they told me, no, it’s psychological because they raised your [calorie intake].... They didn’t believe me until they found me, fainted.... That’s what annoys me, that they don’t trust you. (Danielle) - I was connected to the nasogastric tube again… and I said, this is not the reason I came here. I came so I could learn to live with [anorexia]…. And then they didn’t want me to study [in university], they told me it was a mistake, that I’m not allowed to study, that I need to focus on surviving. I told them that if they think survival is my end [purpose], I prefer to die now. (Emily) - The specifics, they don’t relate to in [the ward]…. The specifics of the individual experience that you are having as a person. They take you as like, you know, you have the symptoms here, you all have them”. (Zoe) - I was connected to the nasogastric tube again… and I said, this is not the reason I came here. I came so I could learn to live with [anorexia]…. And then they didn’t want me to study [in university], they told me it was a mistake, that I’m not allowed to study, that I need to focus on surviving. I told them that if they think survival is my end [purpose], I prefer to die now. (Emily) |  |
| Kezelman et al.,^42^ | | - …people keep telling us ‘you’re sick, you’re sick’ and then you don’t feel anything so you’re like, are you sure?…[but] having this tube especially, it’s like this is what’s needed to make my body function like I’ve put it into a state where my heart physically can’t cater for my body. (P8) - …essentially this place isn’t about getting better…psychologically, just… physically…. (P8) - Whether that tears you down more emotionally…I don’t see the logic in it…When I come out, emotionally [I’ll] be in the same place. I’m not getting the help that I wanted by starving myself in the first place. (P8) - I don’t think it’s individualised in here…. they have their formula and they just put everyone on it…[but] everyone’s problems here are completely different…I don’t think it will be as successful as they think, it doesn’t deal with the psychological or like anything. (P10) |  |
| Smith et al.,^43^ | | - It is assumed that every single thing we say is an eating disorder. Yes sometimes it is but people genuinely do have likes and dislikes. (Participant11) - I feel I am out of the loop and I find that it makes me feel really anxious … everyone is deciding what is happening and nobody is asking me.(Participant 18) |  |
| Thabrew et al.,^44^ | | - ‘I didn’t really feel people were thinking about my feelings’ (P2) - “I would deﬁnitely have appreciated being included more. Maybe asked; I mean, I don’t really know how to ﬁx the problem, I just know that I didn’t appreciate being told what to do and not being included.” (P2). |  |
| MacDonald et al., ^46^ | | - When you then … when you come more out on the other side, then you are able to see that what they did back then, at least some of it, has helped to you still being here. - Um … the worst experiences they’ve definitely been this being strapped tight and then just lying alone. - It is an assault whether it’s been done few or many times. |  |
| O’Connell^47^ | | - Theme Being diagnosed - Theme: Hospital restrictions and a pathologised identity |  |
| **Meta theme 2: Restrictive practice—Living in a “safety bubble”** | | | |
| **Source** | | **Extracts** |  |
| Malson et al.,^37^ | | - Barbara: [T]hat was my total identity. Like I remember at one point in hospital I wasn’t actually able even to read a book or do anything like that because, um, for the internal dialogue of um:well I have to be one hundred per cent anorexic and produce anorexic behaviour at all times.(T14A) - Julie: Everything I say he [a doctor] just like, he laughs at you kind of thing. Like you say one thing and he just laughs and he goes: Oh it’s not her talking, it’s the illness. (T7A)   Elizabeth: I also think talking down to us, like everything is your anorexia and having everything you do analysed as anorexic pattern is very destructive. / Int:mm / I know that they are desperate to find why people are ill and to challenge everything because of that, but there are ways of doing it. (T13UK) |  |
| Colton & Pistrang^38^ | | - ‘It’s a place for hell . . . you’re stuck in here and you can’t get out, you can’t do anything’. (P17) - ‘I think sometimes it’s reassuring because you know what’s going to happen, you know especially around meal times. You know that breakfast is going to be at 8 o’clock and you’re not sat there worrying like when it’s going to be.’ (P9) - ‘I think at the moment freedom is a very dangerous thing for me, ‘cause I don’t know how much damage, I mean people say at the moment you are your worst enemy . . . half of me would say if I was really honest with myself . . . you know, I can’t really control myself and the other half would say I’m fine. Just let me go.’ (P5) - ‘When they’re more encouraging and supportive it makes me want to try harder and when they’re more forceful it makes me always want to pull against and try harder at doing the wrong things.’ (P9)   ‘Some days I just think, just do everything that’s required . . . But then other days I’m just battling against this place.’ (P2) |  |
| Boughtwood & Halse^39^ | | - Nurses still accuse you of trying to vomit, or exercise in the bathroom and, some girls do that but it’s really annoying when you’re not doing it . . . And then, and you think oh well, maybe I should be, heh heh. (Amanda) - He said he had a lot of experience with eating disorders even though he looked about 10, heh, heh, no about 20 or something, like straight out of College sort of thing. So I was, I was really sceptical about like how much experience [in treating anorexia] he’d had. (Sophie) |  |
| Long et al.,^40^ | | - Participant 9 said: “[If there is a delay] everyone can get a bit anxious, asking what’s happening”. - Participant 4 said they: “… just remember thinking how scary it all was”. - Participant 3: “ … all we do, eat and sit and drink, eat and sit and drink”. |  |
| Eli^41^ | | - There is something really good about the fact that there’s an organized diet, that there’s some sort of certainty…. Things are very clear. And it spares you this engagement, a certain part of the engagement that used to exist at an obsessive level. (Alon) - It was a little lab like that, that you could be inside…. A lab in the sense that it was very sterile, it was – very very exact and measured conditions, and – you knew that you, it’s not like the real world, so it eased [our burden]. (Grace) - I didn’t want to leave, I didn’t want to leave, no one wanted to leave… as difficult as it was, there were many difficult things, but – but it was sort of a greenhouse. (Tali)   Even though I feel that I progress a lot and such, I’m really sick of this.… It’s very very difficult for me, this distance from [my daughter], from my home…. Being here, eating this diet, I have no strength anymore, I’m tired already. (Vered) |  |
| Kezelman et al.,^42^ | | When you don’t have a choice there comes a point where you stop worrying about it. (P6)  It makes you not want to cooperate because they don’t really want to understand. (P9)  worried that once I’m discharged…it’ll be more difficult cause I’ll be at a higher weight than I feel comfortable even though it’s healthy. (P5) |  |
| Smith et al.,^43^ | | You don’t have to control it anymore and you can give over that control … it feels as if you are in that stage where you can’t make any decisions … so it is nice to have other people take over. (Participant 17)  It was very scary thinking if I come into treatment I have to hand over all control the eating disorder gave me. That made me feel very unsafe. (Participant 3)  You think can I cope but as you actually manage without using eating disorder behaviours it is so empowering … I can take a normalised control over my well-being and it is me in charge here. (Participant 3)  It is a bit of a safety bubble … You notice when you go out on pass it is so much harder … no one is going to sit and make you eat. (Participant 4)  Sometimes you are needing to speak to somebody and you can’t speak to them … they have to see the new admissions before they see anybody but I have issues as well. (Participant 2)   - I got worse in there because I was given too much choice … Here it is more structured … sometimes getting your choice taken away is better, it shows you it is not all bad. (Participant 16) - I feel I am out of the loop and I find that it makes me feel really anxious … everyone is deciding what is happening and nobody is asking me. (Participant 18) - You become dependent on it … you feel it is your safe place almost. I am almost afraid to be here now because I have become quite attached. (Participant 6) - You go from being an inpatient in here to going out and maybe seeing someone once a week … there is very little support out there … (Participant 1) - I find it difficult to distinguish … what is me and what is the eating disorder … a lot of what my treatment has been is actually finding my own identity. (Participant 3) - I am getting character in myself again and finding a personality and it is all coming back to me who I want to be. (Participant 15) - I think that one to ones we have with our therapists are very important because they help to tackle individuals’ problems. They personalise it for you which I think is necessary. (Participant 5) |  |
| Thabrew et al.,^44^ | | ‘I was in hospital, away from my friends, away from my regular life and doing something that caused a lot of mental stress’ (P3)  ‘It kind of became your safe haven’ (P6)  ‘In hospital, obviously you have to have every last scrap off your plate’ (P1)  ‘I wasn’t allowed to do anything’ (P6)  ‘I kind of felt I was being punished the whole time’ (P4)  ‘Being forced to eat just made me want to restrict more the minute I got out’ (P2)  ‘It was quite relaxing as I felt as though it wasn’t my responsibility for eating the food’ (P9)  ‘(I was) too settled that I was scared to leave’ (P6)  ‘Being forced to eat just made me want to restrict more the minute I got out’ (P2)  ‘It didn’t make me like food any more. It deﬁnitely made me angry’ (P2)  ‘(I was) really stressed about missing school’ (P6) |  |
| Solhaug & Alsaker^45^ | | I`ve debated with myself, worried a lot about the discharge from the hospital, when I´m going home to friends and family again. This forever ongoing pondering lead to a breakdown, and I of course didn`t manage to call the hospital (page 4) |  |
| MacDonald et al., ^46^ | | and it becomes something like an escape, here and now. It is as if it is such a primal instinct that emerges in you. You feel like a hunted animal and you cannot get your more human reasoning into it at all. You resort to that reptile brain, and just feel … you think of ways out and ’escape’. All the time you are hunted somehow.  like the consequence was that if you don’t cooperate, um … then it’s like yes, um … then you get it [nasogastric tube feeding] anyway, then you’re just belt restrained.  That it was the only way that it was for my anorexia to say to me, well … you cannot do anything. Now, it’s okay that you get food because you don’t have the power right now. So, it was really a struggle with myself; that I was expected to take some control, too; and that I should also go against the anorexia. And I just couldn’t at that time. So yes, I think it made me a little dependent on having some involuntary treatment around me, because otherwise it wouldn’t have been okay to eat at all.  It … was just like … the tube directly into the stomach and then … you don’t have to taste anything, you don’t have to smell anything, you don’t have to like you know um, take a stand on anything, um. And that, I think, was somehow extremely relieving. |  |
| O’Connell^47^ | | - Being an inpatient for 8 months had disconnected me from ‘the real world’. I had previously wanted to recover for the sake of my personal relationships and job, but over time these lost their motivational influence. Rather than being immersed in ‘normal life’, I had been in a unit where anorexia was ever present – in other patients, in the treatment programme, in my daily interactions. I was used to anorexia being the standpoint from which I related to others, and the way that I understood myself. I had also been exposed to a new, ‘other’ anorexia, which was more than my own self-starving. It was an anorexia that was bound up with clinical activity and involved being really sick – multiple hospital admissions; concerned doctors; deathly low weights; being detained; physical complica-tions; bed rest; threats of tube feeding. (p. 270) - The construction was reflected in the preventative rules on the SEDU. These rules, such as having to be observed post-meal and not being allowed free access to water, were designed to prevent ‘deceitful’ behaviours such as water loading, hiding food and vomiting after meals. Often, it was the very existence of these rules that made me aware of the behaviours. (p. 272) - During the first few weeks I got used to the hospital routines and what was expected of patients. In some respect I enjoyed eating and was grateful that I ‘had to’, but I worried that this proved the fraudulence of my anorexia. Aware that a patient in a SEDU should be not wanting to eat, as appeared to be the case for other patients, I kept these concerns to myself. (p. 269) |  |
| **Meta theme 3: Myself others and “a similar demon”** | | | |
| **Source** | | **Extracts** |  |
| Colton & Pistrang^38^ | | - ‘ . . . you can talk to them [patients] so much easier than what you can when you’re out of here. Do you know what I mean, you can talk about anorexia just as you can talk about Coronation Street . . . whereas at home, that issue would be totally avoided and I would not even talk about it.’ (P10) - ‘ . . .A lot of people come in here and it’s sort of like who’s the best anorexic.’ (P8) - ‘ . . . it broke my heart because I liked her so much I didn’t know how she could do that [cutting] to herself and I just thought I like her so much better than I like myself so what am I saying by not doing it to myself?’ (P3) |  |
| Long et al.,^40^ | | Participant 11 claimed: “ … to see someone eating what I’ve got in a normal manner is very beneficial … It doesn’t often happen, but some people do eat with us”.  Participant 6: “ … they [other patients] watch each other quite a lot … Who can be the slowest?”   - Participant 11 explained that when watching how others eat a comparison was often made between others’ behaviour and one’s own: “You might see someone doing something, and think, God if I don’t do that, surely I’m not anorexic!” |  |
| Eli^41^ | | - [g]etting there, and sitting in groups, and hearing people talking about things that you’re also going through – there’s something very powerful in this, in this sense of ‘I’m not alone’… [we] feel like, we’re all dealing here with a similar demon, and there’s some sense of shared destiny. (Alon) - I always said that when you’re sitting at the dining hall and you’re spreading a bit of your cheese on the edge of the plate… a person who has an eating disorder will notice and understand it. And that is what I loved so much there, that everyone spoke my language…. Beforehand, I felt so alone, because I felt that no one understood what I was feeling, no one understood what I was saying, no one thought like I did. (Grace) - Emily explained that she could reconsider her own reality through interacting with other patients: [Y]ou see the frustration in seeing an amazing, lovely girl, where there’s nothing, nothing bad to say about her, and she’s just ruining her life over nonsense…. And you suddenly get what other people who talk to you are going through. (Emily) - When you reach a condition that’s relatively healthy and fine and you’re halfway there… suddenly a girl who weighs 20 kilos shows up… I don’t want to see it…. It’s not that it’s the sick side [of me], it’s like – it’s the side I never had. So why do I need to get acquainted with it? (Natalie) |  |
| Kezelman et al.,^42^ | | - It gives you comfort knowing that you’re not the only one…I feel like everyone’s sort of against you in a way, apart from the other girls ‘cause they’re all in the same boat. (P7) - It’s not awkward to talk about [to them] and you don’t feel self conscious or embarrassed at saying that you have a problem or that you need help. (P6) |  |
| Smith et al.,^43^ | | - Nobody is looking at you or judging you … they know exactly what you are going through … it is just almost understood. (Participant 17) - It is really good in terms of being able to hear how other people have gotten over the drive to exercise and how they have managed to eat certain foods. (Participant 3) - It is hard for people when they come in and saying I was in before … maybe that means I am not such as bad anorexic and I need to go home and start relapsing. (Participant 14) - They took us to the pictures … a normal thing to do … nice to know there is life outside. (Participant 18) |  |
| Thabrew et al.,^44^ | | ‘It was quite nice to have other people that kind of understand what you are going through and could relate to what was happening’ (P1)   - ‘It takes some of the pressure off if everyone has to do the same’ (P8) - ‘Seeing what they did kind of gives you ideas about being sneaky’ (P1) - ‘I was kept in for much longer than the other girls’ (P5) - ‘It was hard watching her have like less calories than me’ (P6) - ‘I saw other people that were thinner than me and it made me feel like I had failed at my eating disorder’ (P2) - ‘Since we’re all eating in the same room, you’re experiencing everyone else’s troubles’ (P6) - ‘It was quite nice to have other people that kind of understand what you are going - through and could relate to what was happening’ (P1) |  |
| O’Connell^47^ | | - All the other girls have been loads skinnier than me. Some of them have been in [general] hospital because they’ve been that underweight. And then there’s me (Diary, 10th October 2006). - The first few days of my second admission I was utterly disoriented and as I began eating, overwhelmed by the sensation of food in my stomach. The culture on the unit was different from how it had been previously, due to there being some patients who were under section and (more or less openly) not ‘complying’. Their presence underlined to me the possibility of being on the SEDU as an involuntary patient. I found the idea that, in contrast to them, I was choosing to be in treatment distressing:” Lauren spoke about feeling undeserving and confused about why she’s here i.e. feels ‘normal’ and ‘too big’ etc [. . .] expressing much distress at ‘choosing’ to be here, much guilt after and during eating” (medical notes, SEDU, 13th July 2007). (p.171) |  |
| **Meta them 4: I am not “just another anorexic”** | | | |
| **Source** | | **Extracts** |  |
| Malson et al.,^37^ | | - Jacqui: It’s sort of like speaking to him [a doctor] is like bashing your head up against a wall. / Int: right / Because everything you say is part of the disease. No matter what it is, / Int: right / it’s part of the disease . . . And you’re like: I’m a person. There’s a personality in here you know? It’s not just, / int: mm/ you know I’m not just anorexic kind of thing / Int: right, yeah / which is really tough. (T6A) - Julie: Everything I say he [a doctor] just like, he laughs at you kind of thing. Like you say one thing and he just laughs and he goes: Oh it’s not her talking, it’s the illness. (T7A) - Polly: I thought he [a psychiatrist] was pigeon-holing me. / Int: right, as an anorexic? /Yeah, yeah. And all anorexics think this and do this / Int: right/so you must be like that . . . I didn’t trust any of his advice / Int: right / because I felt he was telling me about what, um, you know, your classic anorexic ought to do. / Int: mm/ And I always felt well he doesn’t know me. So / Int: right / he’s just seeing the illness part of me. He’s not taking me as a whole. (T8UK) - Simone: Within the first 2 days I said: I have, this is a crock of shit. I said this man [a doctor] doesn’t know me, he hasn’t come up and said to me: what are your interests, this this and this./ Int: mm/ And he’s judging me by what’s written down and these measurements, that kind of thing. (T5A) - Clare: I question whether this is the right sort of treatment for me because I feel that my problems aren’t addressed at all and I’m just um (.) being treated as a case book, study sort of thing, out of a textbook. (T2UK) - Barbara: [T]hat was my total identity. Like I remember at one point in hospital I wasn’t actually able even to read a book or do anything like that because, um, for the internal dialogue of um: well I have to be one hundred per cent anorexic and produce anorexic behaviour at all times.(T14A) |  |
| Colton & Pistrang^38^ | | - ‘Just having the confidence to go up to them [staff] or liking them enough to go up to them and ask them you know, ‘‘Can I have a few words with you please?’’ . . . and the good thing is that they nearly always make time to do that.’(P7) |  |
| Long et al.,^40^ | | - Participant 10 described feeling guilty and confused: “ … guilty, like they’ve forced me … well not forced me … while they are there encouraging me to eat it feels ok. But then they go … and I don’t feel as strong anymore … I just get confused about what I’ve done”. |  |
| Eli^41^ | | - They knew about me much more than I knew about myself… things that even I wasn’t aware of, but that they could see from the outside.... It always gave me a good feeling – that I don’t have to talk and they still know. (Grace) - The specifics, they don’t relate to in [the ward]…. The specifics of the individual experience that you are having as a person. They take you as like, you know, you have the symptoms here, you all have them. (Zoey) |  |
| Kezelman et al.,^42^ | | - I don’t think it’s individualised in here…. they have their formula and they just put everyone on it…[but] everyone’s problems here are completely different…I don’t think it will be as successful as they think, it doesn’t deal with the psychological or like anything. (P10) - Wow, these people…[have] faith in me, I need to have faith in myself. (P8) - Engage in some way, [don’t] just [sit] there, because it makes you feel self-conscious and then - you notice more you’re being watched…. (P6) - I’m just this person lying in this bed and people really don’t know what’s going on with me. (P1) |  |
| Smith et al.,^43^ | | - I feel I am out of the loop and I find that it makes me feel really anxious … everyone is deciding what is happening and nobody is asking me. (Participant 18) - Sometimes you are needing to speak to somebody and you can’t speak to them … they have to see the new admissions before they see anybody but I have issues as well. (Participant 2) |  |
| Thabrew et al.,^44^ | | - “I would deﬁnitely have appreciated being included more. Maybe asked; I mean, I don’t really know how to ﬁx the problem, I just know that I didn’t appreciate being told what to do and not being included.” (P2). - ‘It really frustrated and upset me because they [staff] would talk to my parents, but they never talked to me’ (P2) |  |
| Solhaug & Alsaker^45^ | | - I feel pretty tired of being in- hospitalized. I would like to have outpatient- treatment, but the team around me says it too early and that I must wait to get better. I think it`s difficult to be patient. The hours go by so slow, and I don`t know what to do. (page 5) - “I miss just being me, not at patient in need of help and support”. (Page 5) - “because some of the health staff are very strict, something I find challenging. Some of those who have worked here for several years are not that obsessed be the rules”. - “One of the staff took me out for a car ride, then we walked beside the ocean. It was good to see other things and talk about everyday- themes, as for example, the starry sky”. It was nice to have a day where I could experience myself as something else than a patient in need of support and help. Got a feeling of being something more than an illness. It was a little painful to see how much the ED has taken from me when it comes to quality of life and how damaging it is, but it can be useful to remind myself of this when I feel like giving up the battle to become healthy (Page 5) - I feel pretty tired of being in- hospitalized. I would like to have outpatient- treatment, but the team around me says it too early and that I must wait to get better. I think it`s difficult to be patient. The hours go by so slow, and I don`t know what to do (page 5) |  |
| MacDonald et al., ^46^ | | - Um oh well … for instance … it [AN] has forced me to self-harm, um … it has forced me to run away quite a lot and it has forced me to walk, well, 14 km many times a day and such … there have just been so many … patterns and such things that it has forced me to do, where you really … I didn’t want to self-harm, I didn’t want to walk either because I was extremely tired. But like that … it [the AN] has just forced me to that I had to. |  |
| O’Connell^47^ | | - During these admissions especially, my inner world had become bleak and chaotic. My treatment reflected this, as it became ever more restrictive. The more I adopted the ‘anorexic role’, and the more time I spent under restrictive treatment conditions, the more I was distanced from ‘normal life’. In turn, I gripped more tightly onto anorexia. (p.273) - I felt frustrated when staff made pre-emptive decisions due to my perceived ‘risk’. Taking an example from my last admission, I had submitted to staff numerous requests prior to a meeting, such as being allowed home for leave. At this point, there was an expectation that I would be discharged 11 days later, so I argued that leave would be helpful preparation for going home. During the meeting staff decided that my section would be lifted but that my requests would not be met: “She is not close to recovery [and] is very impulsive [. . .] this unit is not the right place for Lauren, as she is eating, but not doing the psychological work [. . .] take off section today, no leave” (Medical notes, SEDU, 6th January 2009). - I also frequently felt ‘unheard’ and my reasoning invalidated due to my inability to escape an anorexic framing. (p. 274) - I had lost all motivation to pursue a career, had no home, no partner, and was accustomed to living in an institution. In these conditions, seeking to do anorexia well (instead of normal life) made sense. (273 |  |
| **Cross cutting theme 1: More than a single experience** | | | |
| **Source** | | **Extracts** |  |
| Colton & Pistrang^38^ | | Phases and transitions observed across themes: 1. What is this illness that I have? 2. Do I want to get well? 5. Collaborating in treatment vs being treated. |  |
| Boughtwood & Halse^39^ | | I saw it as more officially a problem once it was recognized by Dr F, you know, like medically. He told me you have to do something about it. . . because before that it just seemed like oh, if I just ignore it, it’ll go away. But until then until he actually said you know, you do have a problem, there’s something you have to do, it sort of made it, made it more concrete and that it was a big problem. (Renee) |  |
| Long et al.,^40^ | | Transitions noted across themes in authors analysis |  |
| Eli^41^ | | Transitions observed across themes: a sense of recognition and legitimacy; boundaries vis-à-vis the outside world and ones own illness |  |
| Kezelman et al.,^42^ | | - Proposes phases in treatment. - I just wanted to lose weight to be healthy…[but] it somehow got to a point where I got too far. (P4)   I had no idea that it was having an effect on my heart. (P6)   - …I just have to get better, that’s the only way to…get back to life. (P4) - Whereas for others it seemed to reflect a direct challenge to AN cognitions:   …as much as I think that eating the food…getting fat is going to kill me,…it’s not. (P8)   - Even if your thoughts are telling you this is wrong you still have to do it…hoping that if you - keep eating then the thoughts will go away…but it hasn’t been happening yet…. you get - used to [eating large amounts and] don’t look at what you’re eating anymore. You’re just like - I’ve just got to put it in…. (P7) - A select few did develop rationales to assist with managing this intake, though again this - did not seem to reflect a cognitive shift: - it’s just like medicine…you don’t really view it like normal food. (P8)   Adjustment to the persistence of the physiological sensations (e.g. feelings  …knowing the sooner you finish it and put on weight the sooner you’ll be out of here. (P3) |  |
| Smith et al.,^43^ | | Theme’s “experience of transitions”, “shifts in control” and “process of recovery and self-discovery”. |  |
| Thabrew et al.,^44^ | | Transitions and experiences discussed across all themes. |  |
| Solhaug & Alsaker^45^ | | Theme dealing with changes: The participants seemed to have an ongoing battle within themselves during treatment because of the experienced changes. |  |
| MacDonald et al., ^46^ | | Theme: Changing perspectives |  |
| O’Connell^47^ | | Theme: Repeated admissions: Pursuing anorexia  Transitions noted throughout the manuscript   - Darmon (2017) contends that when someone diagnosed with anorexia enters a hospital institution (such as a SEDU), the institution seeks to reverse the individual’s commitment to anorexia by redefining their behaviours and intentions as pathological and replacing them with ‘healthy’ ones. As the individual gets ‘on board’ with this process and internalises the hospitals perspective, they actively invest in ‘recovery’. Recognising oneself as ‘anorexic’ is a necessary step in this process. For me, this recognition occurred prior to entering treatment. However, being in treatment strengthened this self-view by increasing the scope of my experiences that were defined as pathological. Even previously ‘normal’ experiences were retrospectively understood as ‘anorexic’ |  |
| **Cross cutting theme 2: Making meaning and identity** | | | |
| **Source** | | **Extracts** |  |
| Malson et al.,^37^ | | - Int: Is there anything that the nurses say that kind of, or do, that makes you feel more like putting weight on or eating more?   Alice: . . . just knowing that there’s a lot more to life than I s’pose eating disorders. / Int: mm/ Cos well you just get to a point where you think that’s your whole life and that’s all it’s gonna be. (T12A)   - Int: Right, okay. And how did that diagnosis make you feel? Clare: (.) I thought he was mad. (T2UK) - Julie: I wasn’t sick before I came here. I was like healthy. I was just like any other normal teenager . . . I feel well like I just think why the hell am I here [in hospital]? (T7A) |  |
| Colton & Pistrang^38^ | | - ‘I don’t want to have anorexia but at the same time I want to be thin and that’s really strange . . . And I can’t win really, like if I eat I feel guilty, if I don’t eat I feel guilty ‘cause I’m disappointing my family . . . ’ (P16) - ‘Some days I just think, just do everything that’s required . . . But then other days I’m just battling against this place.’ (P2) - Theme 5. Collaborating in treatment vs being treated. - ‘ . . . it does control you and you don’t, even though I didn’t see it, looking back now after being in here I have seen that it did control my life and that’s how I ended up here . . . ’ (P10) - ‘I think for me it numbs a lot of my emotions, it protects me from feeling all that . . . ’ (P1) - ‘It’s like a monster . . . like this big thing with claws wrapped around you . . . and it’s like you. |  |
| Boughtwood & Halse^39^ | | - There’s a lot of, you know, bitching from the girls that goes on about the, you know, staff and that sort of thing, um. . . There is an ‘us versus them’ mentality though, like [the doctors] want me to put on [a certain amount of weight] by Wednesday and um I can’t believe it, and you know. Yeah it’s hard to explain but, there is a real ‘I’ll do it [gain weight] just to make them happy so that I can get home’. Ah, rather than ‘they think that it’s best that I put on this amount of weight, and they know what they’re doing because they’re medical professionals, so I guess it is best for me’. (Renee) - I’ll do it [gain weight] to make them happy so I can get home’. (Renee) - I drank about 4 liters of water. And then I started to lose the feeling in my hands and my feet and after that night I just was, that was my rock bottom sort of thing, like that. After that night I just did, I played by the rules ’cause I just couldn’t, I just was sick of the illness. (Madeline) - You don’t feel much different when it’s that low. Like they say you could go into cardiac arrest or whatever, but it doesn’t feel like that, you just feel normal. (Rita) - Interpretation: Carrie sought to comply with the discourse of the perfect patient. As she explained: ‘That time. . .I tried to do everything’. This response, however, triggered other problems. To gain weight quickly, Carrie ate copious quantities of chocolate. Her distress about her anticipated weight and her actual gain reduced her to tears and she would cry herself to sleep. For the doctors, the intent of weight gain is to facilitate recovery. Carrie’s intent, however, was to escape the clinic. To achieve this goal, she must produce a credible performance for her doctors of the ‘perfect patient’ who wants to recover and will do what is necessary to achieve this goal. For this reason, Carrie can only allow herself to cry at night because she cannot let the doctors see that she is unhappy about gaining weight. (page 88) |  |
| Long et al.,^40^ | | - Participant 3 stated: “I don’t understand how I am meant to be feeling”. - internal battle was described wherein individuals experienced conflicting thoughts which lead to frustration and confusion. Participant 9 described the difficulty of having these opposing sides: “… one side hears, the other side doesn’t. The anorexic part of me finds it really hard, but the well side of me feels proud of me”. |  |
| Eli^41^ | | - Just like you, you know, worked really hard, [and] with blood, sweat, and tears got those two degrees of yours, [then] I’m not worthy of this title, I didn’t spend enough on it… not enough blood, not enough tears, not enough suffering. (Vered) - I told my mom I didn’t want to eat, so I would come [to the ward] as thin as possible… because what if they don’t accept me? What if I gained a kilo? (Tali) |  |
| Kezelman et al.,^42^ | | - You want to be healthier but you still want to feel confident, so the word healthier doesn’t really mean much anymore…because healthier just means less self-confidence…healthier is what’s making you feel bad…. (P7) - [rapid weight gain] really breaks down someone’s self-esteem, which is essentially what the problem has originated from. So then you’re sort of creating a bigger problem whilst making the problem better…. (P8)   …knowing the sooner you finish it and put on weight the sooner you’ll be out of here. (P3)   - Wow, these people…[have] faith in me, I need to have faith in myself. (P8) |  |
| Smith et al.,^43^ | | - I want to get better but I still have the negative thoughts … it is still difficult. I still struggle. (Participant 19) - I am getting character in myself again and finding a personality and it is all coming back to me who I want to be. (Participant 15) |  |
| Solhaug & Alsaker^45^ | | “It was a little difficult to look at myself in the mirror, lately this have been difficult, difficult to see and feel that my body is changing”.  “The despise of bodily curves are chasing me now as I gain weight just gets more hurtful to accept. Painful thoughts of my body are consuming me”.  “I hope we`ll do something as a group today, it helps me handle the days here better and it feels meaningful”. (page 5)  “I believe that the treatment will be easier when I get more responsibility, more possibilities to go outside and experience more freedom”.  “It´s important to remind myself that I am me, not an ED”.  Mostly the grief and ambivalence that were experienced during treatment was expressed by the participants: “Grieving that I let go, and haven´t quite seen the meaning of it today. It´s easier to turn to the ED and not deal with this, but if I´m going further with this and want answers, I have to deal with it.” “I´m mourning over that I have to let go of the ED, because I haven’t seen the meaning of it today.” (page 6)  I`m so tired of having an ED, it`s not a life. (page 7)  This has to be the last time, the ED can`t take any more years from me, it has taken too many already. (page 7)  Sometimes I get afraid and wonder about the meaning of life and the feeling that I´m throwing the life I´ve gotten away. Such thoughts usually come and are most intense at difficult days with little to do when I have much time alone to think. As it was earlier this week; got a strong urge to act destructively/escape by previous strategies, such as self- harming and taking cold showers, but managed to communicate with my contact who works here to derive this by playing cards. I experienced a useful and positive conversation with this contact (page 5) |  |
| MacDonald et al., ^46^ | | Hmm … I think now I’m able to look at it a little more objectively, um … because it’s something else when you’re exposed to it than when you look at it from the outside. Um, but from the outside then I would be able to rationalize that I was is situations where there was simply no other option than that.  I realize that it’s myself who coerces me to like do the told things that the eating disorder … all that it involves, right? I feel it like … enormously tiring and hard and I feel it is … not at all as if I have a life. Not a good life, not very much joy and that is only because I myself live out the actions and do what the eating disorder like … yes, is about.  So, I also think it’s a bit about that I … that I … I have gotten this self-care, um … and dare to take [use] it and treat myself properly. |  |
| O’Connell^47^ | | - Eventually, I realised that the longer I was in and out of treatment, the further the walls would close in. Wanting something different, I tentatively opened up in my mind to the idea of letting go of anorexia. I was discharged for the final time with a strict plan dictating immediate admission to a psychiatric unit or general hospital should my weight drop below an agreed range. I initially maintained my weight, but after around 3 months I began to binge eat uncontrollably. The years that followed involved a painful and turbulent struggle to come to terms with a rapidly changing body and identity as I was no longer classified as ‘anorexic’. (p275) - Thinking of myself as ‘anorexic’ felt like a presumptuous overstatement of my weight loss. Further, maintaining food restriction was like walking a tight rope; at any moment I could slip, lose control of my eating and gain weight. (p. 267) - My life was very limited and monotonous, my mood persistently low and I never laughed anymore. Yet, I simultaneously did not want to stop restricting food. I was captivated by weight loss and unable to imagine living without this exciting ‘thing’ in my life. I relished the secrecy and privacy; I had something that was mine and that no one else could touch. The private knowledge that I was successfully starving felt like a reassuring, comforting presence and nothing else really mattered, so long as I was losing weight. Torn between staying in my pseudo-protective state of starvation, and an increasing sense of guilt for the ‘proper life’ I was not living, I was highly ambivalent. (p.268) - My diaries from this time allude to the two subjective positions that I shifted between; one was that my anorexia was pretend (I was not anorexic at all), and the other was that I was not a ‘good’ anorexic (I was not anorexic enough). The fact that I’m not trying to get out of eating butter when I eat a meal here means I’m not trying hard enough. I was never a good anorexic in the first place because surely then the butter would be harder? I’m a fraud case, I’m not anorexic (Diary, 14th October 2006). The tube functioned as a ‘personal and public signifier of [my] anorexia’ and crucially enmeshed with my identity (Halse et al., 2005: 11). - In addition, the tube signified to me a particular patient ‘type’; the ‘bad anorexic patient’. ‘Bad patient’ is a construction of anorexia that overlaps with ‘serious anorexia’ and denotes someone who is defiant, non-compliant and refuses food at all costs. It is reflected in clinical discourse. In hospital-settings, those diagnosed with anorexia are generally assumed to be ‘non-compliant’ and ‘difficult’ (.P272) |  |
